# Hereditary Thrombophilia Testing Among Hospitalized Patients: Is It Warranted?

**DOI:** 10.7759/cureus.24855

**Published:** 2022-05-09

**Authors:** Omar K Abughanimeh, Rosalyn I Marar, Mohammad Tahboub, Anahat Kaur, Ayman Qasrawi, Mouhanna Abu Ghanimeh, Timothy Pluard

**Affiliations:** 1 Hematology and Medical Oncology, University of Nebraska Medical Center, Omaha, USA; 2 Internal Medicine, University of Nebraska Medical Center, Omaha, USA; 3 Pulmonary and Critical Care Medicine, Tulane University School of Medicine, New Orleans, USA; 4 Hematology and Medical Oncology, Albert Einstein College of Medicine - Jacobi Medical Center, New York City, USA; 5 Internal Medicine/Hematology Oncology, University of Kentucky, Lexington, USA; 6 Gastroenterology, Sanford USD Medical Center, Sioux Falls, USA; 7 Hematology and Oncology, Saint Luke’s Hospital, Kansas City, USA

**Keywords:** venous thromboembolism (vte), testing for thrombophilia, thrombosis, inherited thrombophilia, hereditary thrombophilia

## Abstract

Background

Hereditary thrombophilias (HTs) are a group of inherited disorders that predispose the carrier to venous thromboembolism (VTE). It is estimated that 7% of the population has some form of HT. Although testing for HT has become routine for many hospitalized patients, knowing when to order the tests and how to interpret the results remains challenging. In the United States, there are no clear guidelines regarding testing for HT. We conducted a study to evaluate the utilization of HT testing among hospitalized patients to examine its impact on immediate management decisions and overall cost burden. In addition, we discuss the common reasons for healthcare providers to order these tests and review the data behind these reasons in the literature.

Methodology

A retrospective analysis of 2,402 patients who underwent HT testing between February 1, 2016, and January 31, 2018, was conducted. Eligible patients had at least one HT test ordered during hospitalization. The primary outcome was to determine the incidence of positive actionable tests. A positive actionable test was defined as a positive result that changed the anticoagulation intensity, type, or duration. Patients with a history of previous VTE, ongoing medical conditions requiring life-long anticoagulation, or unprovoked VTE were considered non-actionable.

Results

Among the 2,402 patients, 954 patients met the inclusion criteria with a mean age of 54 years. A total of 397 (41.6%) tests were ordered for acute VTE, while the rest were for non-VTE conditions, such as stroke, pregnancy complications, peripheral artery diseases, and others. Only 89 positive tests were actionable (14% of the positive tests, and 9.3% of the total ordered tests). There was a statistically significant association between increasing age and having both a positive test result (p = 0.006) and an actionable test (p = 0.046). The total cost of ordering these tests was estimated to be $566,585.

Conclusions

HT testing in the inpatient setting did not alter management in many cases and was associated with increased healthcare costs. The decision to order these tests should be individualized based on the clinical scenario.

## Introduction

Arterial and venous thrombosis are common in hospitalized patients. Virchow’s triad describes the three factors that lead to thrombosis, namely, hypercoagulability, stasis, and endothelial injury [1,2]. Thrombophilia is defined as any condition that increases hypercoagulability, which can be acquired or inherited (hereditary) [3]. Hereditary thrombophilias (HTs) are common and prevalent in 7% of the population. It can be caused by mutations in factor V Leiden, mutations in prothrombin 20210, protein C and S deficiencies, antithrombin deficiency, and high levels of homocysteine [1,3].

HT increases the risk of venous thromboembolism (VTE) three to twenty-fold compared to the general population [1]. Although testing for HT is a routine practice, knowing when to order testing and how to interpret the subsequent results can be challenging as the topic is controversial and without much supporting evidence [1,4]. In the United Kingdom, it is estimated that 30,000 tests are done annually to screen for HT with a total cost of 15 million Euros [3]. Due to this financial burden, the British Committee for Standards in Haematology (BCSH) released guidelines in 2010 which recommended against indiscriminate testing for HT in unselected patients presenting with the first episode of VTE [5]. These guidelines highlighted that the management in the acute setting should be the same regardless of HT status. These guidelines mentioned that testing for HT in selected patients with a strong family history of unprovoked recurrent thrombosis may influence management; however, the same guidelines stated that “It is not possible to give a validated recommendation as to how such patients should be selected” [5]. In the United States, there are no clear guidelines. The American Society of Hematology (ASH) released the “Choosing Wisely Campaign” in 2013, which recommended against testing for thrombophilia in VTE associated with major transient risk factors, such as prolonged immobility, surgery, or trauma [6].

The National Institute for Health and Clinical Excellence (NICE) published its newest guidelines in 2020, which recommended against testing for thrombophilia in patients with a prolonged course of anticoagulation or provoked VTE [7]. However, testing for antiphospholipid antibodies can be considered in patients with unprovoked VTE if there are plans to stop anticoagulation or if they have first-degree relatives with VTE. Finally, they recommended against routine thrombophilia testing in patients with first-degree relatives with a history of VTE and thrombophilia.

Our study aims to evaluate the clinical utility of HT testing in the inpatient setting and its concurrent costs. This article was previously presented as a meeting abstract at the 2018 ASH Annual Meeting on November 29, 2018.

## Materials and methods

Study design

We conducted a retrospective study in Saint Luke’s Health System (SLH) in Kansas City, MO. SLH consists of four hospitals, namely, Saint Luke’s Plaza, Saint Luke’s East, Saint Luke’s North, and Saint Luke’s South. Saint Luke’s Plaza is the home of Marion Bloch Neuroscience Institute which is a tertiary referral center for patients with stroke in the Kansas City metro area and Mid America Heart Institute which is a comprehensive cardiac center that serves as a referral center in the area for different cardiovascular diseases. Moreover, it serves as one of the main teaching hospitals for the University of Missouri-Kansas City (UMKC). All study activities were reviewed and approved by the SLH Institutional Review Board. A waiver of patient informed consent was obtained as our study was retrospective in nature.

Study population and data collection

Our study population included patients 18 years or older who underwent any HT testing from February 1, 2016, to January 31, 2018. In total, 2,402 patients with HT tests ordered were identified. Of these, only 954 had testing completed during the index admission. Clinical data were collected regarding study patients’ demographics, hospitalization course, HT tests results, management, and duration of anticoagulation. The hereditary and acquired thrombophilia labs that were reviewed included activated protein C resistance, antithrombin III level, factor V Leiden mutation, factor VIII level, homocysteine level, protein C level, protein S level, prothrombin 20210 mutation, and antiphospholipid panel. Differentiation between provoked and unprovoked VTE was based on a chart review.

Study outcomes

The primary outcome was the proportion of patients with at least one actionable positive test. Actionable is defined as test results that altered anticoagulation intensity, type, or duration. Secondary outcomes included the proportion of positive tests, proportion of each HT test, total cost of inpatient thrombophilia testing, where the test was done (inpatient unit or emergency department (ED)), and ordering providers (ED, hospitalist, consultant).

Statistical analysis

Descriptive statistics were used to summarize patient demographic and clinical characteristics. Fisher’s exact test and Chi-square test were used to determine associations between actionable test results and patients’ clinical and demographic findings. A p-value of <0.05 was considered statistically significant.

## Results

Patient demographics and clinical characteristics

Initially, 2,402 patients with HT tests ordered were reviewed. Of these, 1,448 patients did not have testing during hospitalization and were excluded. The final analysis included 954 patients. The mean age was 54 years, and 56.5% of the cohort were females. Overall, 82.6% of patients were white, and 12% had a family history of previous VTE. Patient demographics are outlined in Table 1.

**Table 1:** Demographic and clinical characteristics of patients. VTE: venous thromboembolism

		Total number	VTE group	Non-VTE group
Age group	18–35 years old	123 (13%)	46	77
	36–55 years old	389 (41%)	176	213
	>55 years old	442 (46%)	175	267
Gender	Male	415 (44%)	165	250
	Female	539 (56%)	232	307
Race	White	788 (83%)	327	461
	African American	152 (16%)	62	90
	Hispanic	12 (1%)	7	5
	Asian	2 (<1%)	1	1
Family history	Yes	113 (12%)	53	60
	No	841 (88%)	344	497

Test ordering characteristics

**Figure 1:**
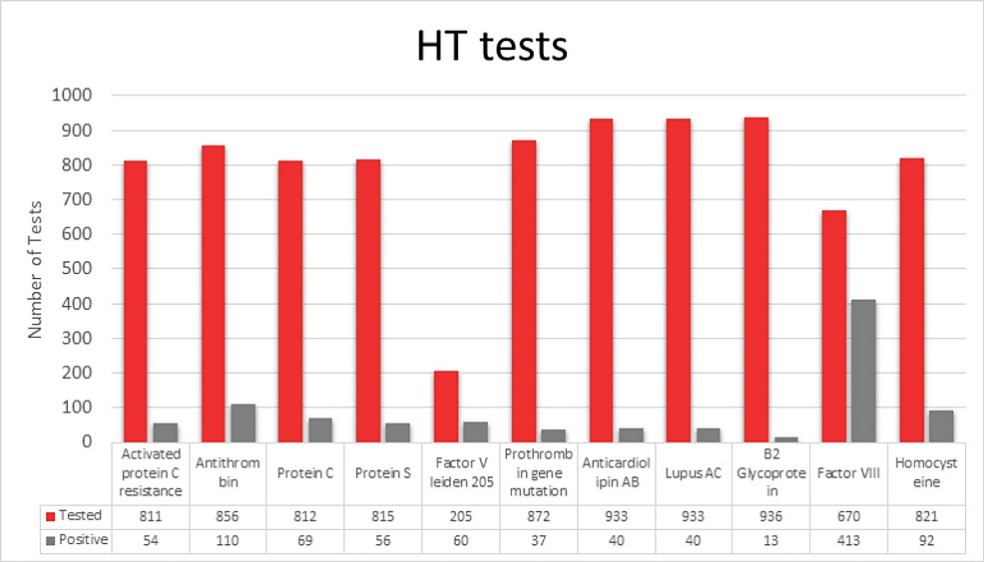
Number of positives in hereditary thrombophilia tests obtained. HT: hereditary thrombophilia

Of all tests ordered, 6.4% were obtained by ED physicians, 43.1% were ordered by hospitalists, and 50.5% were ordered by consultants, with only 4.2% ordered by hematology. Overall, 41.6% of tests were obtained following a diagnosis of VTE, which included pulmonary embolism (PE), deep vein thrombosis (DVT), or both DVT and PE at the same time. Further, 58.4% were obtained following non-VTE events, with stroke being the most common reason. Other causes included pregnancy complications, cardiac thrombus, cerebral vein thrombosis, central retinal artery occlusion, and other peripheral vascular diseases. The most ordered tests included anticardiolipin antibody, lupus anticoagulant, and beta 2 glycoprotein; however, in the majority of cases, a full panel was sent, as shown in Figure 1.

Among all the included patients, 66% (634) had at least one positive test. Overall, 14% of the positive tests and 9.3% of all ordered tests were found to be actionable. Most of the actions were either recommending lifelong anticoagulation or starting anticoagulation in patients with strokes based on the test results. As mentioned above, patients who had medical conditions requiring life-long anticoagulation, a history of previous VTE, or unprovoked VTE were considered nonactionable as the majority of them will require anticoagulation life-long regardless of the test results.

Hereditary thrombophilia testing outcomes and associations

**Figure 2:**
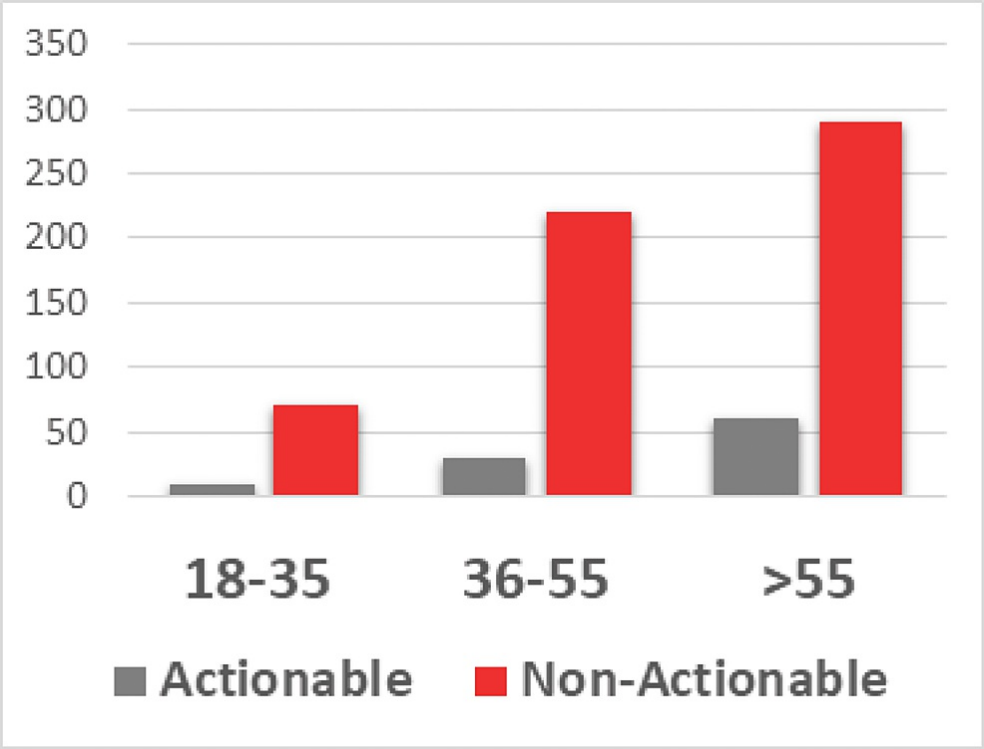
Analysis of positive tests by age group.

There was a statistically significant association between increasing age and having both a positive test (p = 0.006) and an actionable test (p = 0.046) result, as shown in Figure 2.

**Figure 3:**
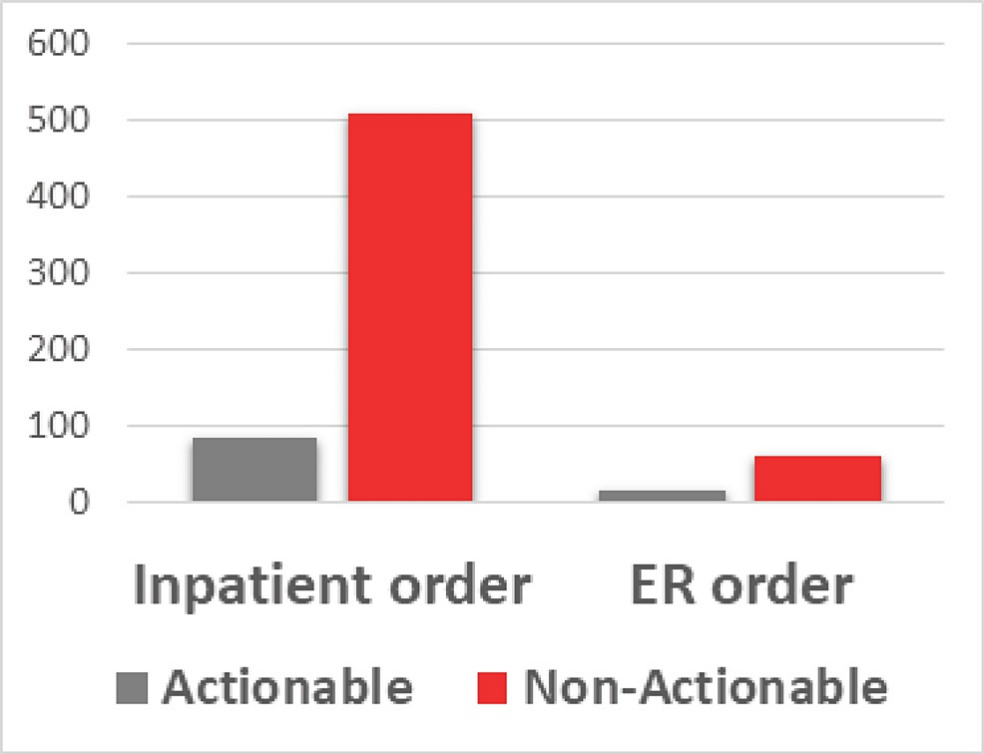
Analysis of positive tests by ordering setting.

There was a statistically significant association between ordering tests in the inpatient setting (rather than the ED) and having an actionable test result (odds ratio (OR) = 0.36; 95% confidence interval (CI) = 0.18-0.74; p = 0.004), as seen in Figure 3.

There was no association between an actionable result and gender (p = 0.123), race (p = 0.451), family history (OR = 1.77; 95% CI = 0.97-3.24; p = 0.061), and diagnosis of VTE (OR = 0.97; 95% CI = 0.61-1.15; p = 0.89).

Cost of hereditary thrombophilia workup

The total cost of ordering these tests was estimated to be $551,218, and the cost of a subsequent hematology consultation was $15,376, leading to a total cost of $566,585 during our study period. This was based on price estimates listed in Table 2.

**Table 2:** Prices of hereditary thrombophilia tests. Ig: immunoglobulin

Test	Cost in US $
Activated protein C resistance	$18.92
Anticardiolipin IgG	$31.42
Anticardiolipin IgM	$31.42
Beta 2 glycoprotein 1 IgG	$31.42
Beta 2 glycoprotein 1 IgM	$31.42
Anticardiolipin antibodies	$31.42
Factor VIII assay	$22.10
Homocysteine	$20.83
Protein C activity	$17.08
Protein S activity	$18.92
Prothrombin gene mutation	$65.69
Antithrombin	$14.63
Factor V Leiden	$230
Hematology oncology consult	$139.70

## Discussion

The first HT condition was described by Egberg in 1965 when he discussed the inherited risk of VTE in patients with a deficiency in antithrombin III [8]. In 1981, Griffin et al. [9] reported an increased risk of VTE in patients with decreased protein C levels, which was followed by similar findings in patients with decreased protein S and mutations in the factor V Leiden gene [10-12].

With these discoveries, testing for HT became common in unselected patients and their relatives in the 1980s-1990s without evidence to support its clinical utility [5]. As a result, multiple committees recommended limiting these tests [5-7]. Despite these recommendations, thrombophilia testing continues to be conducted routinely [4].

Meyer et al. performed a retrospective study on 1,314 patients with documented VTE and assessed the number of patients who had thrombophilia tests ordered. A total of 315 patients had thrombophilia testing performed, but only 31 (10%) patients met the criteria for thrombophilia testing in their institution by having at least one strong thrombophilic risk factor (defined by a history of VTE before 50 years of age, family history of VTE, or recurrent VTE) [13].

Studies have demonstrated that testing of 5,000-10,000 women with proven personal or family history of VTE is required to prevent one death from PE [3]. Hence, is it worthwhile to order these tests frequently? Here, we aim to summarize the controversy regarding ordering these tests during hospitalizations.

First, it is a common belief that HT can predict the risk of VTE recurrence and thus prevent further episodes. However, this statement can be inaccurate. Baglin et al. [14] performed a prospective study where 570 patients who had VTE were followed over a course of two years (patients with malignancies or antiphospholipid syndrome were excluded). About 85% of the cohort had been tested for HT. The cumulative recurrence was 11% after two years. The incidence of recurrence was the highest after an unprovoked VTE (19.4%) and the lowest in a post-surgical VTE. However, recurrence rates were not related to having a positive HT test or not (hazard ratio (HR) = 1.5 [95% CI = 0.82-2.77]; p = 0.187). Similarly, the recurrence rates were not different in patients with unprovoked VTE regardless of their HT testing status. This finding was supported by Christiansen et al. [15] who conducted a prospective study in the Netherland to examine the recurrence rate of VTE in patients after the first episode of VTE. The study included 474 patients who were followed up for a mean of 7.3 years. The rate of VTE recurrence was 25.9/1,000 patient-years (95% CI = 20.8-31.8 per 1,000 patient-years), with the highest incidence in the first two years. In this study, 67% of patients had at least one abnormal HT test. However, there was no difference in recurrence between patients who had a positive HT test and those who had a negative workup (HR = 1.4 [95% CI = 0.9-2.2]) [15]. Other studies have also shown that diagnosing HT is not associated with an increased risk of recurrent VTE [1,16]. Furthermore, the American College of Chest Physicians recommended against using primary prevention prophylaxis in asymptomatic patients with HT (Category 1C) [17]. It is interesting to note that even the risk stratification tools that are used to predict the risk of recurrence of VTE such as the DASH score (D-dimer, age, sex, hormonal therapy) and HERDOO2 (hyperpigmentation, edema, or redness in either leg; D-dimer level ≥250 μg/L; obesity with body mass index ≥30; or older age, ≥65 years) score do not utilize HT test results as one of their parameters [18,19].

Another common reason to test for HT is to screen family members. Providers often order an HT panel based on a family history of clotting disorders as it is a common belief that patients who develop VTE and have a positive family history of VTE have an increased risk of having an HT. It is true that family history is a significant risk factor for a first VTE [20]. Nevertheless, a study showed that family history was associated with a higher risk of VTE regardless of other factors, including having HT conditions such as factor V Leiden, prothrombin 20210A mutation, low antithrombin, protein C, and protein S levels [20]. The study authors concluded that a family history is a risk factor for VTE regardless of other risk factors; therefore, it can be more useful to use in risk assessment than HT testing [20]. In addition, van Sluis et al. [21] performed a retrospective study including 314 patients where they found patients with a family history of clotting disorders had a slightly increased risk of having HT compared to patients without a family history (42% vs. 32%, respectively likelihood ratio 1.3 [95% CI = 0.9-2.1]). They concluded that a family history of VTE is not an effective clinical tool to identify patients with HT. Similarly, our study failed to show a statistically significant association between family history and having an actionable test result. Thus, it remains controversial if HT testing will benefit if the patient already has a family history of VTE. An exception to this would be diagnosing a specific HT condition in young females when counseling them about contraceptive methods [22]. Our study did not show an association between having a positive family history and actionable tests.

Further, some may order the tests to guide the management. In this situation, it is important to recall that treating patients with clotting disorders should not be based on HT test results as initiation or duration of anticoagulation should be the same for all patients with acute VTE [1,4,5]. In fact, HT test results can lead to an unnecessary increase in the duration of treatment and subsequent complications. Sarasin et al. [23] followed a hypothetical cohort of 1,000 carriers of factor V Leiden for five years after completing three months of anticoagulation. They found that prolonged anticoagulation led to a significantly higher number of major bleeding episodes than prevented PE events. They concluded that widespread thrombophilia screening after VTE is not justified [23]. Also, it is important to recognize that some of the HT tests can have false readings if ordered at the time of initial presentation. Protein C and S, antithrombin, and lupus anticoagulant can be falsely abnormal in the setting of acute thrombosis, inflammation, or pregnancy [4]. Moreover, the use of anticoagulants, which is very common in hospitalized patients, can cause false-positive results mostly noticed in antiphospholipid antibodies, protein C, and protein S levels [4]. For this reason, patients taking vitamin K antagonists or direct oral anticoagulants should hold these for an amount of time prior to testing for HT. Lim et al. [24] evaluated 152 patients with acute VTE who had 155 HT tests done. Results showed that 46% of the panels had at least one positive result, but most of these results were falsely abnormal as testing was done in the setting of either acute thrombosis or while receiving anticoagulation. Our study highlights that ordering HT tests did not impact immediate treatment in most patients. An exception to this is if high-risk antiphospholipid syndrome is suspected as studies have shown that warfarin has better efficacy in preventing new clotting events in this population [25].

Another reason to order these tests is to help identify the etiology of the clot. Some argue that identifying HT mutations may help in identifying the cause of hypercoagulability [1]. However, it is important to determine whether there is any underlying benefit of obtaining this additional information, and, most importantly, whether this will result in modifying management practices to guide VTE prophylaxis, testing family members, or help determine the cause [4]. Furthermore, interpreting the results of HT tests is difficult. It is widely agreed upon that a positive test result can improve adherence to VTE prophylaxis in patients [4]. Nevertheless, providers need to keep in mind that a negative test result does not equate to low risk of recurrence and those results may not be accurate.

Finally, some may argue that HT workup should be conducted routinely for patients with stroke, myocardial infarction (MI), and arterial thrombosis. Though, at this time, there is lack of evidence to suggest a correlation between HT and arterial thrombotic events [26]. Clinical manifestations of arterial thrombosis, such as ischemic stroke, are believed to occur mainly as a consequence of vessel wall abnormalities, in particular, atherosclerotic lesions in the setting of endothelial dysfunction and ongoing inflammation [26]. The role of thrombophilia in arterial thrombosis is less established, and, currently, no consensus exists to support the clinical utility and cost-effectiveness of thrombophilia screening in arterial thrombosis. Multiple large studies have investigated the relationship between HT and the risk of developing stroke in unselected ischemic stroke patients and failed to find significant associations [27-29]. In a study among women who suffered an ischemic stroke before the age of 45 years, factor V Leiden was not found to be a risk factor [30]. However, De Stefano et al. [31] reported that patients with a documented ischemic stroke at age less than 50 years and without cardiovascular risk factors were more likely to carry the prothrombin G20210A mutation than controls. In contrast, two other large studies observed no significant association between the G20210A mutation and the risk of developing stroke at a young age [32,33]. Douay et al. [34] conducted a study to evaluate antithrombin, protein C, and protein S levels in 127 young adults (<45 years) with ischemic strokes. They found only nine cases of abnormal levels which led to their conclusion that these deficiencies are rare in ischemic stroke. Another study including 120 young patients (age <45 years) with an ischemic stroke or transient ischemic attack found that many of the HT workups would not be accurate during the stroke workup. Initially, the study found 20 cases of decreased protein S, three cases of decreased protein C, and three cases of decreased antithrombin. However, on repeat labs, they found only two cases of protein S deficiency, and no cases of protein C or antithrombin deficiencies [35]. On the other hand, evidence exists to support a relationship between antiphospholipid syndrome and stroke risk. Several studies show that plasma levels of antiphospholipid antibodies were elevated in young adults who suffered a stroke compared to controls [36-39]. In one study, elevated anticardiolipin antibody levels were detected in 26.9% of cases and 18.2% of controls [39]. In addition, patients with elevated plasma levels of antiphospholipid antibodies were more prone to develop a recurrent ischemic cerebral events than those without [37]. Moreover, a study reported a significant association between beta(2)-glycoprotein 1-dependent anticardiolipin antibody and the risk of having ischemic stroke and MI [40]. So, with the exception of antiphospholipid syndrome, there is no strong evidence that having an abnormal HT testing should affect the clinical decision-making, such as determining the agent and length of anticoagulant treatment. Consequently, testing for thrombophilia defects in unselected patient populations with ischemic stroke is not justified.

A limitation of our study includes the retrospective study design, which has the possibility of introducing recall bias because most of our data collection was dependent on prior documentation. Furthermore, we were only able to establish associations among our variables but could not conclude a cause-effect relationship. Lastly, there is no universally accepted definition for the term actionable test. However, our study highlights the need for larger, multicenter prospective studies to establish clear guidelines for HT testing, including patient selection and appropriate timing of testing.

## Conclusions

Our study demonstrated that HT testing during hospitalization had a limited role in changing management and was associated with a significant cost. The decision to order HT tests should be considered following an individualized clinical risk assessment.
